# Predictors of Entrepreneurship Intention Among Students in Vocational Colleges: A Structural Equation Modeling Approach

**DOI:** 10.3389/fpsyg.2021.797790

**Published:** 2022-01-12

**Authors:** Xueshi Wu, Yumi Tian

**Affiliations:** School of Education, Jiangxi Science and Technology Normal University, Nanchang, China

**Keywords:** entrepreneurial intention, entrepreneurial self-efficacy, emotional competencies, subjective norms, entrepreneurial attitude, higher vocational college students

## Abstract

The purpose of this work is to investigate the major drivers behind the entrepreneurial intention (EI) of students from higher vocational colleges in China. Total 424 respondents participated in the survey questionnaire that measured their self-reported responses to five constructs (EI, entrepreneurial self-efficacy, emotional competencies, subjective norms, and entrepreneurial attitude). In addition to this, the equation modeling technique was used to perform the data analysis. The study results highlighted that both entrepreneurial self-efficacy and entrepreneurial attitudes serve as significant predictors of EI. Moreover, the entrepreneurial attitude was found to be a significant mediator of the EI and emotional competencies. Finally, a model invariance was established across the female and male student samples. As a result, this study has put forward some implications regarding the entrepreneurship education of the higher vocational students in China.

## Introduction

The global spread of COVID-19 will have profound implications for the world economy, especially in terms of jobs and economic growth. In the process of economic development, entrepreneurial activities play a vital role. Vigorous entrepreneurial activities will not only create more jobs, protect people’s livelihood, and improve the sustainability of career development, but also accelerate the commercial application of new knowledge and technologies and help the economy achieve innovation-driven and high-quality development (Nowiński et al., 2019). Since 2014, the Chinese government calls for “mass entrepreneurship and innovation” and “optimizing the environment and providing uninterrupted services to increase the proportion of the college students’ employment and entrepreneurship,” to realize the sustainable development of college students’ career ability.

In China, there are two main types of college graduates: general higher education graduates and higher vocational education graduates. According to data from the National Bureau of Statistics of China, the ratio of the scale of vocational education to the scale of general education in 2020 is about 1:1, and its importance is self-evident (National Bureau of Statistics, 2019). However, ordinary higher education graduates are much higher than the entrepreneurial rate of vocational college graduates, and the ratio is 28%:2% (National Institute Of Education Sciences, 2020; Renmin University of China, 2020). Therefore, the study of entrepreneurial in higher vocational colleges is of great value to improving employment quality and national economy.

Entrepreneurship is so important that scholars have begun to explore what are the key factors that drive individuals to become entrepreneurs. It has also made the scholars investigate the factors that contribute to the initial entrepreneurial behavior of the entrepreneurs. According to Ajzen (1991), behavior performance is affected by all those factors that can indirectly affect human behavior, although, intentions control the human actions as humans do not execute all of their intentions (Ajzen, 1985). Furthermore, several research studies have acknowledged the significance of the entrepreneurial intention (EI). For instance, Krueger et al. (2000) reported that reflexive behavior does not drive entrepreneurial behavior, but it is rather intentional in nature. Similarly, Cai and Zhao (2014) stated that only those people can effectively carry out entrepreneurial activities who have strong EI. EI is one of the important factors that contribute to entrepreneurial behavior whereas EI is rare, but such intentions retain for a longer period of time (Noel, 2002). Lüthje and Franke (2003) advocate that the entrepreneurial behavior of the person is significantly determined by EI. Many studies have investigated the influencing factors of EI from different perspectives. Among previous research models, Theory of Planned Behavior (TPB) has the greatest influence. TPB model has been widely concerned and applied in various research fields, especially in the fields of social science, life science and entrepreneurial management, providing a simple model of the main determinants of individual behavior. In order to enhance the predictive ability of TPB model in higher vocational college students’ entrepreneurial behavior, it is very important to explore other important potential variables through further research. Especially since the proximal variables have been well documented, EI studies need to include other novel predictors. At present, some studies on the influencing factors of students’ EI based on TPB model have been mentioned in academic entrepreneurship literature at home and abroad, but their analysis conclusions are different. Therefore, it is of great significance to expand TPB and study the EI of vocational college students and its influencing factors to promote their sustainable career development.

## Literature Review

### Theory of Planned Behavior

According to the Theory of Planned Behavior (TPB), the intention is based on three rational factors (Ajzen, 1991). Attitude toward the behavior (ATB) that represents the positive or negative evaluation held by the person’s self-performance toward a particular behavior is the first rational factor. The second rational factor is the subjective norm (SN), which highlights the individual perception of a specific behavior being affected by the assessment of a significant person or group (e.g., family members, friends, and colleagues). Perceived Behavioral Control (PBC) is the third rational factor and it reflects personal perception regarding the difficulty in enacting a certain behavior. PBC highlights the major differences between the TPB and the Theory of Reasoned Action (TRA) which was proposed in 1967 by Martin Fishbein and Icek Azjen.

Till present, the TPB, which was initially developed by Ajzen (1991) is the most widely used theory to explain EI.

### Entrepreneurial Intention

It is a commonly held notion that Bird (1988) had initially coined the concept of EI. This concept was defined by him on the basis of behavioral intention as the desire or tendency of a person who consciously directs his action or behavior toward the entrepreneurship activities such as launching a new business. He believed that EI must be used to realize inspired entrepreneurial ideas. Several research scholars have used the same approach to define the concept of EI. For example, Thompson (2009) termed EI as the personal belief that the person holds to establish a new enterprise in the future with a specific plan. Rasli et al. (2013) asserted that the EI shows a mind that motivates the person to establish innovative businesses. Hence, the EI represents the mindset that motivates the person to become an entrepreneur or launch a new business. Qian (2007) believes that EI is one of the effective predictors of entrepreneurial behavior.

The paper has adopted the definition of EI from Liñán and Chen (2009) which represents the EI as the key stimulus that drives the entrepreneur’s determination to build a business and be passionate and serious about being an entrepreneur.

### Entrepreneurial Attitude

The primary predisposing factor of EI is entrepreneurial attitude (EA). EA refers to the individual’s expectations and beliefs about the expected results of entrepreneurial behaviors, and associate them with positive or negative results (Ajzen, 1991). As a rule of thumb, the behavioral intention of the individuals becomes stronger as their attitude becomes more positive toward a particular behavior (Lüthje and Franke, 2003). This means that the more a person thinks entrepreneurship is good, the better his EA will be, and therefore, the stronger his intention to start a business. For example, Acuna-Duran et al. (2021), Boubker et al. (2021), Su et al. (2021), and Tehseen and Haider (2021) have confirmed that a person’s attitude toward entrepreneurship is positively and significantly associated with their intention to start a business. Although, different research studies have been conducted in various cultural contexts and economies but most of these studies still show that there is a significantly positive effect of EA on EI (e.g., Utami, 2017; Pejic et al., 2018; Said et al., 2021).

Nevertheless, certain occasional cases contradict the major findings. such as, Velástegui and Chacón (2021) assert that EA is the weakest predictor of EI among students of public higher education institutions in Ecuador, which contrasts with findings in other countries.

The below hypothesis is postulated based on the above arguments:

H1: EA positively influence EI.

### Subjective Norms

It is a fact that SN is one of the most imperative determinants of the TPB. SN refer to an individual’s perception of whether an individual supports a certain behavior in close social environments, such as family, friends, and colleagues, and the degree to which such evaluation affects the individual (Ajzen, 1991). The behavioral intention of the person becomes stronger with the improvement in the SN for a particular set of behavior.

Subjective norm has also been assumed as an important predictor of EI. Consequently, many researchers in China, Slovenia, Malaysia, and other countries have studied the effect of SN on EI, and pointed out that SN has a direct, positive impact on EI (e.g., Utami, 2017; Pejic et al., 2018; Said et al., 2021; Velástegui and Chacón, 2021). Although, there are certain exceptional studies such as Doanh and Bernat (2019). A TPB was applied by them to survey the EI of students in Vietnam. The results revealed that there is no direct impact of the EI on SN, but there is a significant and indirect impact of the SN on EI through PBC, EA, and ESE. Fernandez-Perez et al. (2019) performed a study in Spain, and they also indicated that there is a positive and direct influence of SN on ESE and EA, but there is an indirect effect of SN on EI. Moreover, SN mainly influences EI through ESE and EA (Liñán and Chen, 2009).

Based on the above arguments, this study has examined both indirect and direct impacts of the SN to thoroughly understand the role of the SN from different perspectives. The following hypotheses are proposed to examine the possible effect of SN:

H2: SN positively influence EI.
H3: SN positively influence EA.

### Entrepreneurial Self-Efficacy

Self-efficacy has been defined by Bandura (1978) as the individual belief regarding his abilities to execute the planned behavior. Therefore, the confidence level of the person to launch a new business with his skills and abilities can be termed as entrepreneurial self-efficacy (ESE).

Bird’s model (1988) had been further modified by Boyd and Vozikis (1994) by including the concept of self-efficacy in that model. It was claimed by them that ESE can influence EI through three different factors such as role models, social support, and previous career.

A number of research studies have reported a positive impact of ESE on business start-up intention (e.g., Liñán and Chen, 2009; Kautonen et al., 2015; Utami, 2017; Bartha et al., 2018; Pejic et al., 2018; Velástegui and Chacón, 2021). In other words, EI is directly related to the level of ESE. Therefore, this study postulates the below hypotheses:

H4: ESE positively influence EI.
H5: SN positively influence ESE.

### Emotional Competency

Emotional competency (EC) is defined by Goleman (1998) as a “learned capability based on emotional intelligence which results in an outstanding performance at work.” Furthermore, Baron (2008) documented that entrepreneurship also includes an emotional component, since there is a possible effect of emotions on cognitively processed information and consequent behavioral tendencies. The TPB theory only takes into account the cognitive characteristics, but that is not sufficient since there is an explicit role of emotional component in entrepreneurship (Cardon et al., 2012).

Several studies report that there is a significant role of EC in shaping the EI, which has a positive and direct relationship with the ESE and EA, but it indirectly affects the EI through these variables (e.g., Fernandez-Perez et al., 2019; Huezo-Ponce et al., 2021; Velástegui and Chacón, 2021). Additionally, Chien-Chi et al. (2020) further proposed that EC can be divided into two dimensions, namely social-emotional competency and personal affective competence. The EI was positively impacted by the social-emotional competency, but the positive impact of personal affective competence on EI was partially supported or not supported at all. It means that the persons who are better able to evaluate, regulate, and monitor their own and other’s emotions are in a better position to perceive higher organizational support and act in an entrepreneurial manner (Padilla-Meléndez et al., 2014).

As a result, it can be inferred that the cognitive abilities are promoted by the greater EC, which motivates the individuals to choose to start their own businesses. But the real practical action in this regard is still a question since there is too little research that confirms whether the EC facilitates the EI.

The below additional hypotheses are postulated based on these arguments:

H6: EC positively influences EI.
H7: EC positively influences EA.
H8: EC positively influences ESE.

This research has constructed a new model based on the previous research studies, emotion–cognitive perspective, and TPB. The newly constructed model is also used to ascertain the effects of cognitive factors (ESE, SN, and EA) as well as EC on the EI of vocational college students.

## Research Methods

### Survey and Sampling Technique

The surveys are classified into two different sections including hypotheses and theoretical background. In the theoretical background section, different demographic-related questions are raised to obtain demographic information of the respondents such as majors’ field, age, gender, etc. While, in the other section that has been adopted from the previous studies, different questions are postulated to facilitate the respondents to share their perspectives on the EI, EA, SN, ESE, and EC.

This study has chosen higher vocational colleges in China as the research sample. Such research samples are relatively famous for entrepreneur-focused studies (Liñán and Chen, 2009). Website access was provided to the students from higher vocational colleges in Jiangxi, China, who had agreed to participate in our research study. The purpose of the website access was to provide the participants with a questionnaire focused on the factors that influence the EI of higher vocational college students. Moreover, as a part of the research ethics, all study participants were informed in advance regarding the study purpose and research questionnaire. This allowed the participants to either accept or refuse to answer the questionnaire within 10–15 min during or after completing the questionnaire (*N* = 424). Table 1 presents the demographic information of the respondents.

**TABLE 1:** Demographic information of respondents.

Demographic variables		*F*	%
Gender	Male	189	44.6%
	Female	235	55.4%
Grade	Freshman	111	26.2%
	Sophomore	236	55.7%
	Junior	77	18.2%
Major field	Humanities and social sciences	144	33.9%
	Natural sciences	280	66.1%
Origin of student	Rural	308	27.4%
	Urban	116	72.6%

### Procedures

A structural equation model (SEM) has been used in this study to test the association between postulated hypotheses. Besides this, the analysis process is based on three major steps. Firstly, the reliability of variables is evaluated using Cronbach’s alpha test. Secondly, confirmatory factor analysis (CFA) has been applied to not only test the empirical validity of each measure and overall research model. Thirdly, the path coefficients of all proposed relationships in the conceptual framework have been estimated through the structural equation model. Furthermore, the variance across the groups and parameters of the model have been tested with the help of a multisample procedure (Byrne et al., 1989). Finally, the AMOS 24.0 and SPSS 26.0 were used to process the data collected through the study questionnaire.

### Measurement Instruments

**FIGURE 1:**
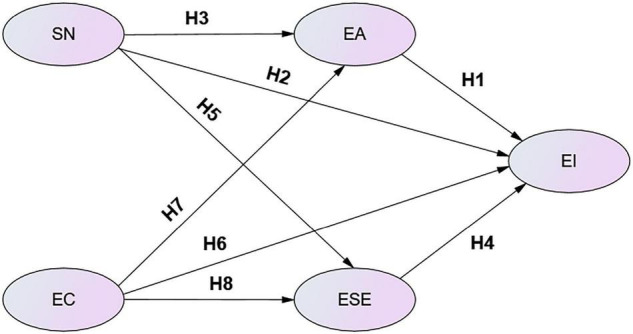
Conceptual framework. SN, subjective norms; EA, entrepreneurial attitude; ESE, entrepreneurial self-efficacy; EI, entrepreneurial intention; EC, emotional competency.

Since the authors did not find any existing instrument suitable for this study, a research model (Figure 1) was developed by the researchers. All variables have been measured through previously validated instruments to construct the study questionnaire. Additionally, each item was measured on a 5-point Likert scale ranging from 1 (totally disagree) to 5 (totally agree). During the actual test, the questions were translated into Chinese to allow the participants to accurately understand the meaning of all questions designed in the questionnaire.

#### Entrepreneurial Intention

Five question items had been borrowed from the scale by Liñán and Chen (2009) to measure the EI. The major reason for adopting the question items from Liñán and Chen (2009) is that these constructs had confirmed the high reliability with Cronbach’s alpha value of 0.946 such as “My professional goal is to become an entrepreneur” and “I have got the firm intention to start a firm someday” and so forth.

#### Entrepreneurial Attitude

Similar to EI, the EA is also measured with the help of five questions being borrowed from Liñán and Chen (2009). Furthermore, these constructs also showed high reliability of Cronbach’s alpha of 0.909 such as “Being an entrepreneur implies more advantages than disadvantages to me” and “If I had the opportunity and resources, I’d like to start a firm” and so forth.

#### Subjective Norms

Three question items were adopted from Kolvereid (1996) and Liñán and Chen (2009) to measure the SN. Their study also confirmed the high reliability of these constructs with Cronbach’s alpha of 0.864 such as “If I decided to create a firm, my closest family would approve of that decision” and “If I decided to create a firm, people who are important to me would approve of that decision” and so forth.

#### Entrepreneurial Self-Efficacy

Entrepreneurial self-efficacy was assessed through 5 question items borrowed from De Noble et al. (1999) and Liñán (2008). Both research studies had confirmed the high reliability of the constructs (Cronbach’s alpha of 0.931) such as “I can work productively under continuous stress, pressure, and conflict” and “I can originate new ideas and products” and so on.

#### Emotional Competency

Emotional competency was also evaluated with the help of 3 different question items borrowed from Goleman (1998) and Cherniss and Goleman (2005). Both of the studies indicated the high reliability of the constructs with Cronbach’s alpha of 0.914 such as “I acknowledge the needs of other people to progress, and I like to foster their capabilities” and “I can make use of effective means of persuasion” and so forth.

## Results

### Descriptive Statistics

Table 2 represents the descriptive statistics of the constructs.

**TABLE 2:** Descriptive statistics.

	Mean	SD	Skewness	Kurtosis	Cronbach’s alpha
SN	3.53	0.73	−0.27	0.89	0.86
EA	3.61	0.76	−0.64	1.03	0.91
ESE	3.55	0.72	−0.44	1.04	0.93
EI	3.48	0.78	−0.44	0.81	0.95
EC	3.69	0.59	−0.61	2.48	0.91

*SN, subjective norms; EA, entrepreneurial attitude; ESE, entrepreneurial self-efficacy; EI, entrepreneurial intention; EC, emotional competency.*

The descriptive statistics of the constructs show that all means/averages, excluding EI which is 3.48, are higher than the midpoint of 3.50, ranging from 3.69 to 3.48. In addition to this, the standard deviations (S.D) range from 0.78 to 0.59. The skew index ranges from –0.27 to –0.64, whereas the kurtosis index ranges from 2.48 to 0.81. The kurtosis and skew indices are acceptable and indicative of univariate normality (Teo et al., 2009). Hence, the research data for this work is assumed to be sufficient for structural equation modeling.

### Test of the Measurement Model

Confirmatory factor analysis (CFA) was applied to test the quality of the measurement model. Additionally, the significance of the individual item loadings (*t*-values) was examined to establish the model fit. The use of fit indices from different categories have been suggested by Hair et al. (2006). The efficiency with which the observed data is reproduced by the proposed model is measured by absolute fit indices. The absolute fit indices are similar to the indices of parsimony except that the former also considers the complexity of the model. Furthermore, the relevance between specific model fit to the alternative baseline model is assessed with the help of incremental fit indices. Several statistical tests, including comparative fit index (CFI), the root mean square error of approximation (RMSEA), χ^2^ statistic, Tucker-Lewis index (TLI), and standardized root mean residual (SRMR), have been used in this study. The chi-square divided by the degrees of freedom (χ^2^/df) must not surpass the value of 3 for a model to be considered as a good fit (Carmines and McIver, 1981), as well as both CFI and TLI should both exceed the value of 0.95 (Hu and Bentler, 1999). Hair et al. (2006) asserted that both SRMR and RMSEA should not exceed 0.08 to be considered adequate.

The factor loadings of all items on the constructs in the measurement model are displayed in Table 3. All parameter estimates are significant (*p* < 0.05) as highlighted by the *t*-value higher than 1.96 or critical ratio (CR). Additionally, the *R*^2^ values are above 0.50 for all variables except EC1 (*R*^2^ = 0.41), which indicates that each indicator has explained more than half of the variance in the latent variable. Nunnally and Bernstein (1994) point out that the alpha values, which range from 0.94 to 0.95 are considered high. This shows that there is an internal consistency in the items. Lastly, sufficient model fit for the measurement model was reported (χ^2^ = 427.321, χ^2^/df = 2.387, GFI = 0.915, AGFI = 0.890, TLI = 0.963, CFI = 0.968, RMSEA = 0.057, SRMR = 0.033). The adequacy of the measurement model shows that the items are reliable indicators of the hypothesized constructs. As a result, it facilitates the tests of the structural associations in different models to proceed.

**TABLE 3:** Convergence validity.

		Unstd.	S.E.	*t*-value	*p*	Std.	SMC	CR	AVE
EA	EA1	1.000				0.710	0.504	0.948	0.797
	EA2	1.183	0.070	16.827	***	0.858	0.736		
	EA3	1.056	0.067	15.669	***	0.807	0.651		
	EA4	1.171	0.071	16.495	***	0.848	0.719		
	EA5	1.256	0.074	16.883	***	0.866	0.750		
EC	EC5	1.000				0.922	0.850	0.950	0.859
	EC4	0.917	0.049	18.642	***	0.868	0.753		
	EC1	0.609	0.043	14.136	***	0.643	0.413		
EI	EI2	1.000				0.912	0.832	0.957	0.792
	EI3	0.843	0.035	23.983	***	0.823	0.677		
	EI4	0.969	0.030	31.969	***	0.928	0.861		
	EI5	0.891	0.037	23.899	***	0.822	0.676		
	EI6	0.944	0.034	27.601	***	0.876	0.767		
ESE	ESE1	1.000				0.753	0.567	0.951	0.862
	ESE2	1.034	0.058	17.868	***	0.834	0.696		
	ESE3	0.985	0.053	18.634	***	0.866	0.750		
	ESE4	1.037	0.054	19.301	***	0.894	0.799		
	ESE6	0.978	0.055	17.904	***	0.836	0.699		
SN	SN3	1.000				0.866	0.750	0.952	0.765
	SN2	0.974	0.053	18.217	***	0.839	0.704		
	SN1	0.935	0.055	17.081	***	0.770	0.593		

***denotes p < 0.001; SN, subjective norms; EA, entrepreneurial attitude; ESE, entrepreneurial self-efficacy; EI, entrepreneurial intention; EC, emotional competency.

### Discriminant Validity

Discriminant validity denotes the significant difference or low correlation between the potential variables represented by one or another facet.

The Fornell and Larcker criteria as a measurement, suggested by Fornell and Larcker (1981), is to identify the multicollinearity issues among constructs in this work. The result shows that there are fewer multicollinearity issues among constructs, whereby the square root of AVE (diagonal) is more significant than correlations (off-diagonal) for all constructs in the Table 4 below.

**TABLE 4:** Discriminant validity.

	ESE	EC	EI	SN	EA
ESE	**0.928**				
EC	0.923	**0.927**			
EI	0.826	0.759	**0.890**		
SN	0.585	0.591	0.541	**0.875**	
EA	0.850	0.780	0.882	0.567	**0.893**

*SN, subjective norms; EA, entrepreneurial attitude; ESE, entrepreneurial self-efficacy; EI, entrepreneurial intention; EC, emotional competency. The diagonal elements (bold) are the square root of AVE.*

### Path Coefficient Analysis

The major purpose of the path coefficient is to assess the significance of explanatory variables toward the dependent variable. This work has proposed the directional hypotheses for H1–H8, where *p*-value should be less than 0.05 and *t*-value should be more than 1.657 for hypotheses acceptance as recommended by Hair et al. (2014). Since this criterion was not fulfilled for the three hypotheses of this work, therefore, H2, H5, and H6 were rejected based on Table 5.

**TABLE 5:** Summary of the hypothesis tests.

Hypotheses	Path	Path coefficient	*t*-value	*p*	Results
**H1**	EA → EI	0.675	9.657	***	**Supported**
H2	SN → EI	0.021	0.528	0.598	Not Supported
**H3**	SN → EA	0.102	2.021	*	**Supported**
**H4**	ESE → EI	0.417	2.645	**	**Supported**
H5	SN → ESE	0.019	0.455	0.649	Not Supported
H6	EC → EI	–0.171	–1.015	0.310	Not Supported
**H7**	EC → EA	0.772	12.070	***	**Supported**
**H8**	EC → ESE	0.943	15.829	***	**Supported**

***denotes p < 0.001; **denotes p < 0.01; *denotes p < 0.05; SN, subjective norms; EA, entrepreneurial attitude; ESE, entrepreneurial self-efficacy; EI, entrepreneurial intention; EC, emotional competency; figures in parentheses are the standardized estimates. Supported means the results accept the hypothesis. Not supported means the results reject the hypothesis.

The study results highlight that ESE (β = 0.417, *p* < 0.001) and EA (β = 0.675, *p* < 0.001) both significantly influence the EI, hence supporting H1 and H4. Moreover, SN had a significant effect on EA (β = 0.102, *p* < 0.001), and a non-significant effect on and ESE (β = 0.019, *p* > 0.05) and EI (β = 0.021, *p* > 0.05). These findings support H3 but turn void the H5 and H2. Lastly, a significant effect of EC was reported on EA (β = 0.772, *p* < 0.001) and ESE (β = 0.943, *p* < 0.001). On other hand, EC demonstrated an insignificant effect on EI (β = –0.171, *p* > 0.05). Thus, it can be implied that the students will perceive themselves as more capable of becoming entrepreneurs as a higher degree of EC leads to a more positive EA. These findings of our study support H7 and H8 but reject H6.

### Mediating Testing

The Bootstrap method is an ideal test method to investigate the presence of mediating effect (Preacher and Hayes, 2008). The criterion is when the confidence interval of the estimated indirect effect does not contain 0 then there is a significant mediating effect.

Table 6 demonstrates the test results which confirm that emotional competency affects EI through EA. In other words, it shows that the EA serves as the mediator as there is an insignificant effect of EC on EI, whereas the EC affects the EI through the mediator EA. These results suggest that EI is developed among the students who develop higher EC through positive EA.

**TABLE 6:** The mediating testing result.

Parameter		Estimate	Lower	Upper	*p*	Results
ind1	**EC → EA → EI**	0.675	0.464	0.940	0.001	**Supported**
ind2	EC → ESE → EI	0.509	–0.001	1.293	0.050	Not Supported
ind3	SN → EA → EI	0.081	–0.023	0.216	0.115	Not Supported
ind4	SN → ESE → EI	0.009	–0.048	0.090	0.603	Not Supported
total		1.077	0.982	1.197	0.001	
r1	Ind1/total	0.627	0.439	0.889	0.001	
r2	Ind2/total	0.473	0.018	1.246	0.047	
r3	Ind3/total	0.075	–0.022	0.193	0.116	
r4	Ind4/total	0.009	–0.047	0.081	0.610	
diff1	ind2-ind1	0.166	–0.639	0.802	0.602	
diff2	ind4-ind3	–0.072	–0.192	0.006	0.063	

*SN, subjective norms; EA, entrepreneurial attitude; ESE, entrepreneurial self-efficacy; EI, entrepreneurial intention; EC, emotional competency. Supported means the results accept the hypothesis. Not supported means the results reject the hypothesis.*

### Multigroup Analysis

Entrepreneurship has long been seen as a typically male profession, and women have often found the rewards of starting a business to be less attractive (Shinnar et al., 2014; Nowiński et al., 2019). This is because women think that it is difficult to balance the role of female entrepreneur with that of traditional women. Running a business takes so much effort that female entrepreneurs are often unable to care for their families. Following the previous research, we consider it necessary to test whether the proposed model is invariant when applied to male and female samples.

The purpose of this analysis was to assess the invariance in the proposed model being used for both male and female samples. An initial unconstrained model was constructed to examine whether the pattern of factors and items was feasible across the study samples. A multisample procedure with five consequential hypotheses was applied to consider the final model (Byrne et al., 1989). They are as follows: (a) Measurement weights model, male and female students have equal factor loadings on the structural equation model of EI; (b) structural weights model, male and female students have equal path coefficient on the structural equation model of EI; (c) structural covariances model, male and female students have equal factor covariance on the structural equation model of EI; (d) structural residuals model, male and female students have equal latent variable error equation on the structural equation model of EI; and (e) measurement residuals model, male and female students have equal error term of the observed variable on the structural equation model of EI. Each of the above five models needs to be compared with the unconstrained model, respectively.

The comparison and goodness-of-fit indices for all models in the invariance routine are plotted in Tables 7, 8.

**TABLE 7:** Fitting index of multigroup analysis.

Model	CMIN	DF	*p*	TLI	CFI	RMSEA
Unconstrained	772.674	360.000	0.000	0.939	0.948	0.052
Measurement weights	786.788	376.000	0.000	0.942	0.948	0.051
Structural weights	792.795	384.000	0.000	0.944	0.948	0.050
Structural covariances	820.011	387.000	0.000	0.941	0.945	0.051
Structural residuals	833.925	390.000	0.000	0.940	0.944	0.052
Measurement residuals	877.280	411.000	0.000	0.940	0.941	0.052

**TABLE 8:** Test invariance of multigroup analysis.

Model	Δ CMIN	Δ DF	*p*	Δ TLI	Δ CFI	Δ RMSEA
Measurement weights	14.114	16.000	0.590	0.003	0.000	–0.001
Structural weights	20.121	24.000	0.690	0.005	0.000	–0.002
Structural covariances	47.337	27.000	0.009	0.002	–0.003	–0.001
Structural residuals	61.251	30.000	0.001	0.001	–0.004	0.000
Measurement residuals	104.606	51.000	0.000	0.001	–0.007	0.000

Table 7 indicates that the fitting index is sound. Subsequently, Table 8 highlights that the chi-square difference tests of the measurement weights model and structural weights model were insignificant. While, on other hand, the chi-square difference between the unconstrained model and the other three models (measurement residuals model, structural residuals model, and structural covariances model) was significant. Additionally, the incremental fit indices demonstrate that such change supports the differences that are insignificant as shown by a change of 0.01 or less in the fit indexes (Cheung and Rensvold, 2002). Therefore, we concluded in support of the model invariance across the samples of male and female students.

## Discussion

The main aim of this work was to find out the major drivers behind the EI of college students. The literature review has proposed EI as one of the determinants of entrepreneurial activities. Additionally, it has been hypothesized that EI is estimated by different independent variables including EC, ESE, SN, and EA. The research model (Figure 1) was tested using structural equation modeling techniques, and the results revealed a good fit for the data. Most of the hypothesized associations among the constructs in the research model were supported, accounting for 80% of the total variance in the EI. In the subsequent paragraphs, the study findings are discussed to address the research questions being raised in the introduction section.

The study findings indicate that ESE and EA have a significant and direct association with the EI. The study results confirms that the Chinese higher vocational college students who have a positive attitude to start and run their own companies have a positive EI, consistent with the studies by Said et al. (2021) and Su et al. (2021). It also justifies that the students from Chinese higher vocational college who believe in their abilities to achieve the set targets in the critical conditions are more inclined to have higher EI as supported by the study from Fernandez-Perez et al. (2019).

Subjective norm did not demonstrate a significant impact on EI. Furthermore, the results suggested that there is no mediating effect of SN on EI through ESE or EA. Contrarily, SN displayed a significant effect on EA. Since most of the students in Chinese vocational college lack social experience and do not constitute an independent personality, their attitude toward entrepreneurship is easily influenced by the people close around them, such as friends and family members. However, it is not mandatory that the students will must change their EI in this manner.

Emotional competency also did not demonstrate a significant impact on EI. This shows that EC completely affects EI through the mediator EA as reported by Huezo-Ponce et al. (2021). It also shows that the EI of students in Chinese higher vocational college is not significantly affected, whereas the EC is well-supported. However, when the students display a positive attitude toward entrepreneurial behavior then EC became an important consideration. Therefore, the colleges should not pass on the entrepreneurial knowledge and skills being required by the students, but should encourage students to obtain more pronounced emotional intelligence.

Finally, a multigroup analysis was conducted to verify the invariance of the proposed model in both male and female samples. It was found that there is no significant difference in EI influencing factors between male and female students.

Our study findings not only contribute to the development of entrepreneurship theories but also extend vital inspiration for entrepreneurship role among Chinese students of higher vocational college. It also supports the practice and training of entrepreneurship education in higher vocational institutions and universities. Hence, this work provides an important framework to understand the EI of the Chinese students by exploring the emotional and cognitive factors and their possible interrelationships. Consequently, this work proposes to achieve a rational balance, particularly in the decisions with emotional dimensions.

## Conclusion

This study has explored four major driving factors behind the EI of Chinese students among higher vocational college. This work has put forward research implications for the researchers and educators to understand the major drivers and their antecedents, and how these factors interact to explain the high and low levels of entrepreneurial intent among students. Since, entrepreneurship requires analytical management skills, the EI of the students will increase or at least continue to sustain when universities and other higher educational institutions effectively manage the stated four variables in the competitive learning environment.

## Limitation and Future Research

Despite the significant contributions offered by this study, it has some limitations and unaddressed questions. First of all, this work only looked at expected intentions toward expected behavior, not the behavior itself. Therefore, future studies should be designed to cover time from a longer perspective. Secondly, convenience sampling rather than random sampling was used in this study, and all students came from a single province, which may affect the representativeness and universality of the results. In this regard, it is recommended that future research may compare crossregional students from different parts of the same country or different countries to test the hypothesis of the study, which have different cultural, social norms, and academic backgrounds. Finally, the relevant impact of entrepreneurship education is not mentioned in the current research framework. Ni and Ye (2018) studied the EI of students in Secondary vocational schools in China and pointed out that entrepreneurship education can play the role of a booster and further improve their comprehensive quality and lay the foundation for their future career development. There is a positive effect of entrepreneurship education on EI among students from secondary vocational schools in China. Therefore, in future studies, we will add this variable to enrich our research results.

## Implications

First, a variety of measures are taken to improve students’ EC. On the one hand, the cultivation of EC is incorporated into the compulsory courses of vocational college students, leading students to understand the inner logic of emotions from multiple perspectives such as sociology and psychology, and master appropriate emotional attitudes and emotional skills; on the other hand, build emotions based on the Internet platform General education courses to overcome the shortcomings of lack of professional resources and narrow coverage of offline courses, so that resources can benefit as many vocational college students as possible. Secondly, vocational colleges and governments should attach great importance to cultivating college students’ EA and ESE, and pay special attention to factors related to EA and ESE. The government and schools can promote the advantages of entrepreneurship through courses, hold entrepreneurial activities, social media, and other channels to create a good entrepreneurial atmosphere, such as propagating to students that entrepreneurship has the advantages of solving employment problems, realizing economic independence, realizing their own values and dreams, and making contributions to the society and economy. In addition, from the perspective of SN, families should also provide higher vocational graduates with greater tolerance and support in terms of entrepreneurship. Through these approaches, students’ EA can be changed from bad to good, so that they can form their EI under the influence of follow-up training guidance and entrepreneurial role models. Finally, the government should create a better entrepreneurial environment for college students, such as establishing social entrepreneurship support projects, providing entrepreneurial funds, and providing free entrepreneurial venues so that college students can easily start their businesses.

## Data Availability Statement

The raw data supporting the conclusions of this article will be made available by the authors, without undue reservation.

## Author Contributions

XW: conceptualization. XW and YT: data curation. YT: writing original draft, writing, review, and editing. Both authors have read and agreed to the published version of the manuscript.

## Conflict of Interest

The authors declare that the research was conducted in the absence of any commercial or financial relationships that could be construed as a potential conflict of interest.

## Publisher’s Note

All claims expressed in this article are solely those of the authors and do not necessarily represent those of their affiliated organizations, or those of the publisher, the editors and the reviewers. Any product that may be evaluated in this article, or claim that may be made by its manufacturer, is not guaranteed or endorsed by the publisher.
